# Bt cotton producing Cry1Ac and Cry2Ab does not harm two parasitoids, *Cotesia marginiventris* and *Copidosoma floridanum*

**DOI:** 10.1038/s41598-017-18620-3

**Published:** 2018-01-10

**Authors:** Jun-Ce Tian, Xiang-Ping Wang, Yang Chen, Jörg Romeis, Steven E. Naranjo, Richard L. Hellmich, Ping Wang, Anthony M. Shelton

**Affiliations:** 1 Department of Entomology, Cornell University/New York State Agricultural Experiment Station (NYSAES), New York, United States of America; 2 State Key Laboratory Breeding Base for Zhejiang Sustainable Pest and Disease Control, Institute of Plant Protection and Microbiology, Zhejiang Academy of Agricultural Sciences, Hangzhou, China; 3 College of Agriculture, Yangtze University, Jingzhou, Hubei China; 4 Key Laboratory of Biotechnology in Plant Protection of MOA and Zhejiang Province, Institute of Virology and Biotechnology, Zhejiang Academy of Agricultural Sciences, Hangzhou, China; 5 Agroscope, Research Division Agroecology and Environment, Zürich, Switzerland; 6 USDA-ARS, Arid Land Agricultural Research Center, Maricopa, Arizona United States of America; 7 USDA–ARS, Corn Insects and Crop Genetics Research Unit and Department of Entomology, Iowa State University, Ames, Iowa United States of America

## Abstract

Cabbage looper, *Trichoplusia ni* (Hübner) is an important lepidopteran pest on many vegetable and greenhouse crops, and some field crops. Although there are no commercial transgenic Bt vegetable or greenhouse crops, *T*. *ni* is a target of Bollgard II cotton, which produces Cry1Ac and Cry2Ab. We expand on previous work that examined the effect of Bt crops on parasitoids using Bt-resistant lepidopteran populations as hosts. Cry1Ac/Cry2Ab-resistant *T*. *ni* larvae were used to eliminate host quality effects and to evaluate the direct effects of Bt cotton on the parasitoids *Copidosoma floridanum* (Ashmead) and *Cotesia marginiventris* (Cresson). These tri-trophic studies confirm that Bt cotton had no significant impact on development, success of parasitism, survival and adult longevity of *C*. *marginiventris* when using Bt-resistant *T*. *ni* fed on Bt cotton. Similarly, this Bt cotton had no significant impact on the development, mummy weight and the number of progeny produced by *C*. *floridanum*. Our studies verified that lyophilized Bt crop tissue maintained its insecticidal bioactivity when incorporated into an artificial diet, demonstrating that hosts and parasitoids were exposed to active Cry proteins. The egg-larval parasitoid *C*. *floridanum*, or similar species that consume their entire host, should be considered useful surrogates in risk assessment of Bt crops to non-target arthropods.

## Introduction

The area planted to genetically engineered insect-resistant crops producing proteins from *Bacillus thuringiensis* (Bt) has expanded rapidly since their first commercial production in 1996. Over 53 million hectares of insect-resistant maize and 22 million hectares of cotton were cultivated globally in 2016, and global adoption rates for insect-resistant maize and cotton were 29% and 64%, respectively^1^. Although benefits of Bt crops to pest suppression, grower economics, human health and the environment have been well documented^2–6^, the potential effect of Bt crops on non-target organisms, especially natural enemies that play an important role in pest control, continues to be debated^7–10^.

Most studies have indicated that Bt crops do not pose a threat to natural enemies^11–13^. However, some authors have suggested that Bt crops have negative effects on natural enemies, especially parasitoids^14^. In those studies, Bt-susceptible prey or hosts were used as carriers to transfer Bt protein to natural enemies. Such methods thus do not allow one to distinguish whether the negative effects were due to the prey or host quality or to the Bt proteins^11,15,16^. One of the best ways to eliminate the potential impact of host or prey quality is to use a Bt-resistant host or prey that can develop well on the Bt crop, and thereby transfer a high concentration of Bt protein to the host or prey and eventually the natural enemy^17^.

Cabbage looper, *Trichoplusia ni* (Hübner) (Lepidoptera: Noctuidae) is an agricultural pest that feeds on >160 plant species in 36 families^18^, and is an important lepidopteran pest on many vegetable and greenhouse crops, and some field crops. Although there are no commercial transgenic Bt vegetable or greenhouse crops, *T*. *ni* is a target of Bollgard II cotton, which produces Cry1Ac and Cry2Ab and is widely cultivated in the USA^19^.

We are not aware of a population of *T*. *ni* that has evolved resistance to Bt cotton in the field, but a Bt-resistant *T*. *ni* population was found in a commercial greenhouse that had been routinely treated with foliar sprays of Bt^20^. This Bt-resistant *T*. *ni* strain was further selected on Bt cotton in the laboratory^21^ and has been used in several studies to assess the potential effects of Cry1Ac/Cry2A cotton on natural enemies, including *Coleomegilla maculata* (DeGeer) (Coleoptera: Coccinellidae)^22^, *Chrysoperla rufilabris* Burmeister (Neuroptera: Chrysopidae)^23^, *Zelus renardii* (Kolenati) (Hemiptera: Reduviidae)^24^, *Geocoris punctipes* (Say) (Hemiptera: Geocoridae) and *Orius insidiosus* (Say) (Hemiptera: Anthocoridae)^25^.

*Cotesia marginiventris* (Cresson) (Hymenoptera: Braconidae) is a parasitoid wasp that attacks a wide range of noctuid species, including *T*. *ni*. Adult females usually deposit one egg into a young host larva (first to second instar). The egg hatches and the parasitoid larva develops through three instars by feeding on hemolymph and other tissues, eventually killing the host. The parasitoid larva then emerges from the host to pupate and form a cocoon^26^. *Copidosoma floridanum* (Ashmead) (Hymenoptera: Encyrtidae) is a polyembryonic parasitoid wasp. Adult females attack the host eggs and the polyembryonic egg of copidosomines divides by holoblastic cleavage during host egg-larval development, which results in the formation of large numbers of undifferentiated morulae. The parasitoid larvae hatch during the host’s final instar, rapidly consume the host, and then pupate within the remnant host cuticle^27^. Both of these parasitoids attack *T*. *ni*, which exposes them to Cry proteins when *T*. *ni* feed on Bt crops.

In the present study, we expand on previous works on the effect of Bt crops on parasitoids using Bt-resistant lepidopterans as hosts. Cry1Ac/Cry2Ab-resistant *T*. *ni* was used to eliminate host quality effects and to evaluate the direct effects of Bt (Cry1Ac/Cry2Ab) cotton on these two parasitoids, *C*. *floridanum* and *C*. *marginiventris*. Development, mummy weight, and the number of progeny produced by *C*. *floridanum*, and development, success of parasitism, survival, and adult longevity of *C*. *marginiventris* were evaluated in tri-trophic studies.

## Results

### Bioassay with *T*. *ni*

There were no significant differences in survival of the *T*. *ni* larvae when fed either Bt or non-Bt cotton incorporated diet (Table 1). Development time from pupa to adult was significantly faster on Bt cotton, but this amounted to only 0.3 d out of 8 d total. In contrast, larval development was slightly prolonged on Bt cotton by 0.6 d out of 13 d total (*P* = 0.05). There were no significant differences in any of the other life table parameters (Table 1).

**Table 1:** Impact of Bt (Cry1Ac/Cry2Ab) cotton on life-table parameters of Cry1Ac/Cry2Ab-resistant *Trichoplusia ni*. Data are shown as means ± SE. Number of replications are given in parentheses.

Parameters	Non-Bt Cotton	Bt Cotton	Statistics
Larval survival (%)	95.0	96.0	*χ* ^2^ = 0.65; *df* = 1; *P* = 0.72
Development time (d)			
Larva-pupation	13.0 ± 0.2 (47)	13.6 ± 0.2 (48)	*t* = 1.96; *df* = 93; *P* = 0.05
Pupal stage	7.9 ± 0.1 (41)	7.6 ± 0.1 (42)	*t* = 2.25; *df* = 81; *P* = 0.03
Pupal weight (g)	0.23 ± 0.01 (47)	0.22 ± 0.01 (48)	*t* = 0.64; *df* = 93; *P* = 0.52
Adult longevity (d)	4.5 ± 0.1 (41)	4.6 ± 0.1 (42)	*t* = 0.54; *df* = 81; *P* = 0.59

### Tri-trophic bioassay with *C*. *marginiventris*

Nine to 13 d after parasitism, *C*. *marginiventris* larvae emerged from *T*. *ni* and formed cocoons. Adults emerged 5–7 d later. There were no significant differences detected for any of the life table parameters measured for *C*. *marginiventris* (Table 2).

**Table 2:** Tri-trophic effects of Bt (Cry1Ac/Cry2Ab) cotton on life table parameters of *Cotesia marginiventris* parasitizing Cry1Ac/Cry2Ab-resistant *Trichoplusia ni* reared on Bt cotton or non-Bt cotton incorporated diet. Data are shown as means ± SE; each experimental unit was replicated 10 times.

Parameters	Non-Bt cotton	Bt cotton	Statistics
Development time (days)			
Eggs to cocoons	10.4 ± 0.1	10.2 ± 0.1	*t* = 1.27; *P* = 0.23
Cocoons to adults	6.1 ± 0.1	6.0 ± 0.1	*t* = 0.79; *P* = 0.45
Success of parasitism (%)	96.0 ± 1.6	95.0 ± 2.2	*t* = 0.36; *P* = 0.72
Cocoon-adult survivorship (%)	85.0 ± 2.7	86.0 ± 2.2	*t* = 0.29; *P* = 0.78
Sex ratio (% females)	58.8 ± 2.3	57.0 ± 2.8	*t* = 0.51; *P* = 0.62
Female longevity (days)	9.7 ± 0.5	10.1 ± 0.6	*t* = 0.53; *P* = 0.60

### Tri-trophic bioassay with *C*. *floridanum*

Seventeen to 23 d after parasitism, parasitized *T*. *ni* larvae formed mummies. *C*. *floridanum* adults emerged 9–12 d later. No significant difference was found for any of the life table parameters measured for *C*. *floridanum* between the Bt cotton and non-Bt cotton treatment (Table 3). Similarly, no significant differences were detected for any of the life table parameters for the 2^nd^ generation of the parasitoids (Table 3).

**Table 3:** Tri-trophic effects of Bt (Cry1Ac/Cry2Ab) cotton on life table parameters of *Copidosoma floridanum* parasitizing Cry1Ac/Cry2Ab-resistant *Trichoplusia ni* reared on Bt cotton or non-Bt cotton incorporated diet over two generations. Data are shown as means ± SE. Number of replications are given in parentheses.

Parameters	Non-Bt cotton	Bt cotton	Statistics
Development time (d)			
1^st^ Generation			
Egg-mummy	18.8 ± 0.2 (47)	19.1 ± 0.1 (48)	*t* = 1.45; *df* = 93; *P* = 0.15
Mummy-adult	10.0 ± 0.1 (41)	10.1 ± 0.2 (42)	*t* = 1.02; *df* = 81; *P* = 0.31
Egg-adult	29.2 ± 0.1 (41)	29.5 ± 0.2 (42)	*t* = 1.47; *df* = 81; *P* = 0.14
Mummy weight (g)	0.44 ± 0.01 (47)	0.43 ± 0.01 (48)	*t* = 1.48; *df* = 81; *P* = 0.14
Progeny number	1993.8 ± 67.0 (30)	1957.5 ± 70.8 (30)	*t* = 0.50; *df* = 24; *P* = 0.62
2^nd^ Generation			
Egg-mummy	18.7 ± 0.1 (39)	18.9 ± 0.1 (42)	*t* = 1.17; *df* = 93; *P* = 0.25
Mummy-adult	9.4 ± 0.1 (36)	9.1 ± 0.1 (38)	*t* = 1.46; *df* = 81; *P* = 0.15
Egg-adult	27.9 ± 0.2 (36)	28.3 ± 0.1 (38)	*t* = 1.80; *df* = 81; *P* = 0.08
Mummy weight (g)	0.43 ± 0.01 (39)	0.42 ± 0.01 (42)	*t* = 1.62; *df* = 81; *P* = 0.11
Progeny number	2280.7 ± 57.7 (30)	2197.2 ± 59.8 (30)	*t* = 1.95; *df* = 81; *P* = 0.06

### Bioactivity of Cry1Ac/Cry2Ab in cotton powder, diet and *T*. *ni*

Extracts from Bt cotton leaf powder, Bt cotton incorporated diet and *T*. *ni* larvae fed on Bt cotton incorporated diet were toxic to susceptible *Plutella xylostella* (Linnaeus) (Lepidoptera: Noctuidae) (Table 4).

**Table 4:** Mortality rate of Bt-susceptible *P*. *xylostella* larvae fed cabbage leaves treated with Cry1Ac/Cry2Ab cotton powder, Bt cotton-incorporated diet and Bt cotton-incorporated diet-fed *Trichoplusia ni*. Data are shown as means ± SE.

Treatment	Mortality % (Means ± SE) of susceptible host
Bt cotton leaf powder	44.0 ± 9.27 a
Non-Bt cotton leaf powder	8.0 ± 2.00 b
Bt cotton incorporated diet	34.0 ± 2.45 a
Non-Bt cotton incorporated diet	6.0 ± 2.45 b
*T*. *ni* fed on Bt cotton incorporated diet	32.0 ± 2.00 a
*T*. *ni* fed on non-Bt cotton incorporated diet	12.0 ± 3.74 b
ddH_2_O (Control)	4.0 ± 2.45 b

A total of 50 susceptible *P*. *xylostella* larvae were used in each treatment with 5 replications (10 larvae/replication). Means (±SE) followed by different letters are significantly different (One-way ANOVA, *P* < 0.05).

## Discussion

Studying potential impacts of genetically engineered insect-resistant crops on beneficial non-target arthropods (NTAs) is an important component of environmental risk assessment. Initial studies to support risk assessment are conducted in the laboratory and provide information on whether a high dose of the biologically active insecticidal compound is toxic to the test species^8^. Such laboratory studies need to be carefully designed to provide robust data that contribute to the confidence in the non-target risk assessment^17,28^.

One key element of such studies is to ensure that the test species was exposed to a high dose of a bioactive Bt protein. Herbivores that have consumed a Bt crop, and are then used as prey or host for a natural enemy, is a common method for Bt exposure. The interpretation of the results, however, may be inaccurate. If the herbivore is susceptible to the Bt protein then its health is compromised and there is potential for host/prey-quality mediated effects^11,29^. Effects on the NTA, if observed, could erroneously be interpreted as direct toxic effects of Bt proteins and this misinterpretation has occurred in various tri-trophic studies, especially on parasitoids that are more susceptible to the effects of host quality^15,16^. A more appropriate way to avoid such host/prey-quality mediated effects in tri-trophic study is using Bt-resistant herbivores as a Bt protein carrier^16^.

Cry1Ac/Cry2Ab-resistant *T*. *ni* have been used as a Bt protein carrier in several previous studies demonstrating that Bt cotton does not harm five predatory species including *C*. *maculata* ^22^, *C*. *rufilabris* ^23^, *G*. *punctipes* ^25^, *O*. *insidiosus* ^25^ and *Z*. *renardii* ^24^. The Cry1Ac/Cry2Ab-resistant *T*. *ni* strain has adapted to an artificial diet and consequently survival was reported to be <80% when fed Bt or non-Bt cotton leaves for 7 d^22^. The quality of cotton leaf-fed *T*. *ni* was still sufficient for assessing the potential risk of Bt cotton to predators because larvae were only fed on Bt cotton for 3–4 d. However, the situation is different with parasitoids that are more intimately associated with the host’s development. *C*. *marginiventris* and *C*. *floridanum* need to develop inside the body of *T*. *ni* for ca 10 d and 19 d, respectively. The host quality of Bt-resistant *T*. *ni* that feed on cotton leaves would not be adequate for assessing the potential risk of Bt cotton on parasitoids. To overcome the problem, cotton leaves were lyophilized and incorporated into the artificial diet. The larval survival of *T*. *ni* fed on cotton leaf incorporated diet was >95% (Table 1). Using caterpillars of the Bt-sensitive *P*. *xylostella*, we demonstrated that these diet exposures retained the bioactivity of Cry1Ac/Cry2Ab protein in lyophilized cotton leaves, Bt cotton incorporated diet, and *T*. *ni* that fed on Bt cotton incorporated diet (Table 4). This demonstrates that *C*. *marginiventris* and *C*. *floridanum* were exposed to bioactive Cry1Ac/Cry2Ab protein.

*C*. *floridanum* is a polyembryonic egg-larval parasitoid. Numerous *C*. *floridanum* larvae hatch in the host’s ultimate instar and consume the entire host rapidly. Thus, when hosts feed on Bt crops, *C*. *floridanum* is much more likely to be exposed to Bt proteins than larval parasitoids like *C*. *marginiventris*, which only feed on specific host tissues, and may possibly avoid tissues containing Bt proteins such as the gut^30^. Thus, *C*. *floridanum*, or similar species that consume their entire host, should be considered useful surrogates in risk assessment of Bt crops to NTAs.

Our results confirm that Bt cotton containing Cry1Ac/Cry2Ab has no significant impact on development, success of parasitism, survival and adult longevity of *C*. *marginiventris*. Our results differ from two previous studies using Bt-susceptible herbivores to assess the potential effects of Bt crops on *C*. *marginiventris*. In both these studies, *C*. *marginiventris* were significantly negatively affected when using either Bt-maize fed *Spodoptera littoralis* (Boisduval) (Lepidoptera: Noctuidae)^30^ or Cry1Ab fed *Spodoptera frugiperda* (J. E. Smith) (Lepidoptera: Noctuidae)^31^. In both cases, the adverse effects on the parasitoids were likely caused by the fact that the hosts themselves were sublethally affected by the Bt Cry proteins. Our results agree with a previous study that used Cry1F-resistant *S*. *frugiperda* as hosts to evaluate the potential risk effects, which found that *C*. *marginiventris* was not affected by the Cry1F protein^32^. Our findings also indicate that development, mummy weight and the number of progeny produced by *C*. *floridanum* were not affected by Cry1Ac/Cry2Ab when using Bt-resistant *T*. *ni* as a Bt protein carrier.

To our knowledge, only three other studies have used a Bt-resistant host to evaluate the potential effects of Bt crops on parasitoids. Cry1Ac-resistant *P*. *xylostella* were used in the study with *Cotesia plutellae* (Kurdjumov) (Hymenoptera: Braconidae)^33^, Cry1C-resistant *P*. *xylostella* were used in a study with *Diadegma insulare* (Cresson) (Hymenotera: Ichneumonidae)^34^ and Cry1F-resistant *S*. *frugiperda* were used in the study with *C*. *marginiventris* ^32^. All of these studies demonstrated that these proteins do not harm the parasitoids. Using Bt-resistant hosts, our results further confirm these general finding for Cry1Ac/Cry2Ab and two parasitoids species.

In summary, our tri-trophic studies demonstrated that *C*. *marginiventris* or *C*. *floridanum* are not sensitive to Cry1Ac/Cry2Ab. These results, together with other published literature, demonstrate no adverse effects of Cry1Ac/Cry2Ab-expressing Bt cotton on non-target species outside the target order of Lepidoptera.

## Materials and Methods

### Plants

Seeds of Bt cotton (Bollgard II^®^), producing Cry1Ac and Cry2Ab, and the corresponding non-transformed near isoline Stoneville 474, were obtained from Monsanto (St. Louis, MO). Bt cotton and non-Bt cotton plants were individually grown in 6 L plastic pots in the same greenhouse at 27 ± 2 °C under a light and dark regime of 16:8 h. Cotton leaves were collected and lyophilized (freeze dried) when the plants reached the 12 leaf stage. The lyophilized cotton leaves were crushed by mortar and pestle to obtain a fine powder, which was stored at −20 °C and used in the experiments (see artificial diets below).

### Insects

A Cry1Ac/Cry2Ab-resistant *T*. *ni* strain (GLEN-BGII) was originally collected from commercial greenhouses in British Columbia, Canada, and further selected on Bollgard II cotton foliage in the laboratory^21^.

To detect the bioactivity of Cry1Ac/Cry2Ab, we used a Bt-susceptible strain of the diamondback moth, *P*. *xylostella* (strain G88), which has been continuously reared on an artificial diet since 1988^35^. Second instar *P*. *xylostella* were used for detecting bioactivity of Cry1Ac/Cry2Ab, as described below.

*C*. *marginiventris* and *C*. *floridanum* were obtained from Mike Strand (Department of Entomology, University of Georgia). *C*. *marginiventris* had been maintained on non-Bt maize-fed *S*. *frugiperda* and *C*. *floridanum* had been maintained on original artificial diet-reared *T*. *ni* (strain GLEN-BGII) for several generations before used in the present study.

All insects were maintained in a climatic chamber at 27 ± 1 °C and 50 ± 10% RH, under a light and dark regime of 16:8 h. All experiments also were conducted under these conditions.

### Artificial diet for *T*. *ni*

An artificial diet for *T*. *ni* was used in all experiments because the strain does not perform well on cotton^22^. Briefly, 1 L diet contained 120 g wheat germ, 34 g casein, 10 g cellulose, 8 g Wesson salt mix, 10 g USDA vitamin premix, 2 g sorbic acid, 1 g methyl paraben, 37 mg chlortetracycline, 15 g agar and 800 ml ddH_2_O. The original diet was used to maintain the Cry1Ac/Cry2Ab-resistant *T*. *ni* strain. For the experiments described below, lyophilized Bt or non-Bt cotton leaf powder (5%) was added to the artificial diet.

### Bioassay of *T*. *ni* reared on Bt or non-Bt cotton incorporated diet

The development, pupal weight and longevity of Cry1Ac/Cry2Ab-resistant *T*. *ni* were compared on Bt cotton incorporated diet with those reared on a non-Bt cotton incorporated diet. Newly hatched individual neonates were reared in 30-ml Cometware^TM^ plastic cups (WNA, Covington, KY). Every 4 d, larvae were removed from the cups and transferred to a cup of fresh diet with the same treatment. The survival of *T*. *ni* larvae was recorded daily until pupation. Pupae were collected, weighed and transferred to a new 30-ml cup. Pupae were checked daily until adults emerged. Unfed adults were kept individually in cups and were checked daily until dead. Fifty replications were conducted for each treatment.

### Tri-trophic bioassay with *C*. *marginiventris*

Newly emerged female and male *C*. *marginiventris* adults were paired in 0.5 L clear plastic soda bottles in which the bottom was removed and covered with cotton gauze. Parasitoids were supplied with a 10% sugar water solution-saturated cotton wick. After allowing parasitoids 48 h for mating, ten 4-d old *T*. *ni* larvae reared on Bt or non-Bt cotton incorporated diet were presented to paired wasps for 24 h. Larvae were exposed to parasitoids by placing them in a Petri dish (5 cm diam.) with either Bt or non-Bt cotton incorporated diets.

After the 24 h exposure period, the *T*. *ni* larvae were individually transferred into 30-ml cups with Bt or non-Bt cotton incorporated diets. After parasitoid exposure the larvae were transferred individually into 30-ml cups containing the same diet. Diet was renewed every four days. *T*. *ni* larvae were checked for survival twice per day (0900 and 2100) to record parasitoid cocoon formation. The parasitism rate was recorded based on the number of cocoons that had formed. Cocoons were individually transferred to clear 30-ml cups and checked twice per day (0900 and 2100) until adults emerged. Adults were provided with a 10% sugar water solution-saturated cotton wick and checked twice per day (0900 and 2100) until dead. Ten pairs of *C*. *marginiventris* were utilized for both Bt cotton and non-Bt cotton treatments.

### Tri-trophic bioassay with *C*. *floridanum*

Newly emerged female and male *C*. *floridanum* were allowed to mate for 24 h before 100 females were individually transferred to 100 Petri dishes (15 cm diam.) containing a piece of wax paper with 24 h old *T*. *ni* eggs. Parasitism was observed and parasitized *T*. *ni* eggs were marked by drawing a small circle around them with a pencil and unparasitized eggs were then removed. After parasitized *T*. *ni* eggs hatched, one neonate from each female wasp was randomly selected and the neonates (50 replicates per treatment) were individually transferred into 30-ml cups that contained Bt or non-Bt cotton incorporated diet. Diet cups were renewed every four days. *T*. *ni* larvae were recorded daily until mummies formed, whereupon they were weighed and transferred into a glass tube (2 cm diam., 15 cm height). Mummies were checked daily until *C*. *floridanum* emerged. Thirty mummies were randomly selected from both treatments and progeny were counted. *C*. *floridanum* that emerged from the remaining mummies were used to repeat the test on the next generation in the same manner as described above.

### Bioactivity of Cry1Ac/Cry2Ab in cotton powder, diet and *T*. *ni*

To check for bioactivity of the Bt proteins we used the procedures described by Tian *et al*.^36^. Samples of Bt cotton leaf powder, non-Bt cotton leaf powder, Bt cotton incorporated diet, non-Bt cotton incorporated diet, 6-day old *T*. *ni* that had fed on Bt cotton incorporated diet and 6-day old *T*. *ni* that had fed on non-Bt cotton incorporated diet were used in this experiment. Samples of cotton powder and diet were diluted at a rate of 1:400 and 1:20 (mg sample: µL ddH_2_0), respectively. The samples of *T*. *ni* were homogenized at a rate of 1:10 (mg sample: µL ddH_2_0). Bond-spreader sticker (Loveland Industry, Loveland CO) was added at 0.1% to each sample solution before being applied to cabbage leaf disks (diameter 3 cm). The experimental unit was ten 2^nd^ instar Bt-susceptible *P*. *xylostella* (strain G88) placed on each of the leaf disks inside 30-ml cups. The experiment was conducted as a randomized design with 5 replicates per treatment. Larval mortality was checked after 72 h.

### Statistical analyses

Survival analysis of *T*. *ni* that fed on Bt or non-Bt cotton incorporated diet was conducted using the Kaplan-Meier procedure and Log-rank test. Data on life table parameters of *T*. *ni*, *C*. *marginiventris* and *C*. *floridanum* were analyzed using Student’s *t*-test. Data on bioactivity of Cry1Ac/Cry2Ab were analyzed using a one-way ANOVA and Tukey’s multiple comparison test. Before analysis, all percentage data were arcsine of square root transformed, as necessary, but untransformed means are presented. All statistical calculations were performed with SAS version 9.1 package^37^. For all tests, α = 0.05.
